# 10-(3,5-Dinitro­phen­yl)-5,5-difluoro-1,3,7,9-tetra­methyl-5*H*-dipyrrolo­[1,2-*c*:2′,1′-*f*][1,3,2]diaza­borinin-4-ium-5-uide

**DOI:** 10.1107/S1600536813008027

**Published:** 2013-04-05

**Authors:** Ai-Jun Cui, Yan Wang, Jie He, Xiang Li, Ming-Yang He

**Affiliations:** a Key Laboratory of Fine Petrochemical Technology, Changzhou University, Changzhou 213164, People’s Republic of China

## Abstract

In an effort to discover new potential boron-dipyrromethene (BODIPY) dyes, the title compound, C_19_H_17_BF_2_N_4_O_4_, was prepared from 2,4-dimethyl­pyrrole, 3,5-dinitro­benzaldehyde and boron trifluoride in a one-pot reaction. The BODIPY fragment is nearly planar, with a maximum deviation from the least-squares plane of 0.251 (2) Å, and the benzene ring is inclined at a dihedral angle of 86.8 (6)° to the BODIPY mean plane. In the crystal, pairs of C—H⋯F hydrogen bonds connect neighbouring mol­ecules into inversion dimers, which are linked by further strong C—H⋯F inter­actions, forming a supra­molecular layered array parallel to the *bc* plane.

## Related literature

For the use of related compounds for fluorescence analysis, see: Weiner *et al.* (2001 ▶); Gabe *et al.* (2004 ▶). For the structural characterization of related compounds, see: Euler *et al.* (2002*a* ▶,*b* ▶); Cui *et al.* (2006 ▶). For the synthetic procedure, see: Kollmannsberger *et al.* (1998 ▶).
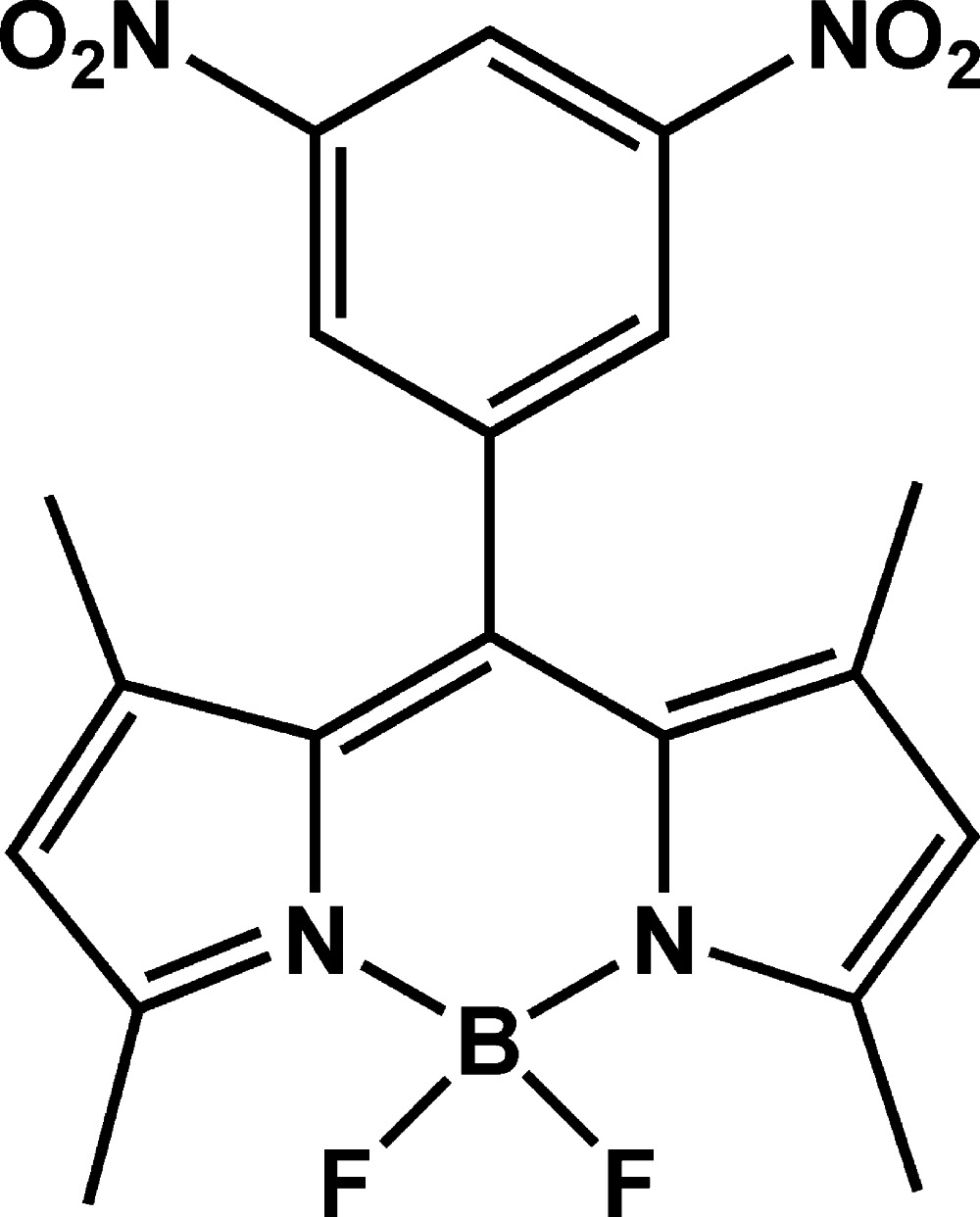


## Experimental

### 

#### Crystal data

C_19_H_17_BF_2_N_4_O_4_
*M* *_r_* = 414.18
Monoclinic, 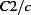

*a* = 29.016 (3) Å
*b* = 9.1763 (9) Å
*c* = 16.8294 (16) Å
β = 121.086 (2)°
*V* = 3837.4 (7) Å^3^
*Z* = 8
Mo *K*α radiation
μ = 0.11 mm^−1^
*T* = 295 K
0.21 × 0.21 × 0.16 mm

#### Data collection

Bruker APEXII CCD diffractometer
Absorption correction: multi-scan (*SADABS*; Sheldrick, 2003 ▶) *T*_min_ = 0.973, *T*_max_ = 0.985
11322 measured reflections
3767 independent reflections
2276 reflections with *I* > 2σ(*I*)
*R*_int_ = 0.035

#### Refinement

*R*[*F* ^2^ > 2σ(*F* ^2^)] = 0.045
*wR*(*F* ^2^) = 0.114
*S* = 1.04
3767 reflections
275 parameters
H-atom parameters constrained
Δρ_max_ = 0.19 e Å^−3^
Δρ_min_ = −0.19 e Å^−3^

### 

Data collection: *APEX2* (Bruker, 2007 ▶); cell refinement: *APEX2* and *SAINT* (Bruker, 2007 ▶); data reduction: *SAINT*; program(s) used to solve structure: *SHELXTL* (Sheldrick, 2008 ▶); program(s) used to refine structure: *SHELXTL*; molecular graphics: *SHELXTL*; software used to prepare material for publication: *SHELXTL*.

## Supplementary Material

Crystal structure: contains datablock(s) I, global. DOI: 10.1107/S1600536813008027/zl2539sup1.cif

Structure factors: contains datablock(s) I. DOI: 10.1107/S1600536813008027/zl2539Isup2.hkl

Supplementary material file. DOI: 10.1107/S1600536813008027/zl2539Isup3.cml

Additional supplementary materials:  crystallographic information; 3D view; checkCIF report

## Figures and Tables

**Table 1:** Hydrogen-bond geometry (Å, °)

*D*—H⋯*A*	*D*—H	H⋯*A*	*D*⋯*A*	*D*—H⋯*A*
C5—H5⋯F2^i^	0.93	2.51	3.307 (2)	144
C13—H13*A*⋯F1^ii^	0.96	2.45	3.287 (2)	146

Symmetry codes: (i) 
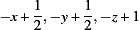
; (ii) 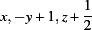.

**Fig. 1.**
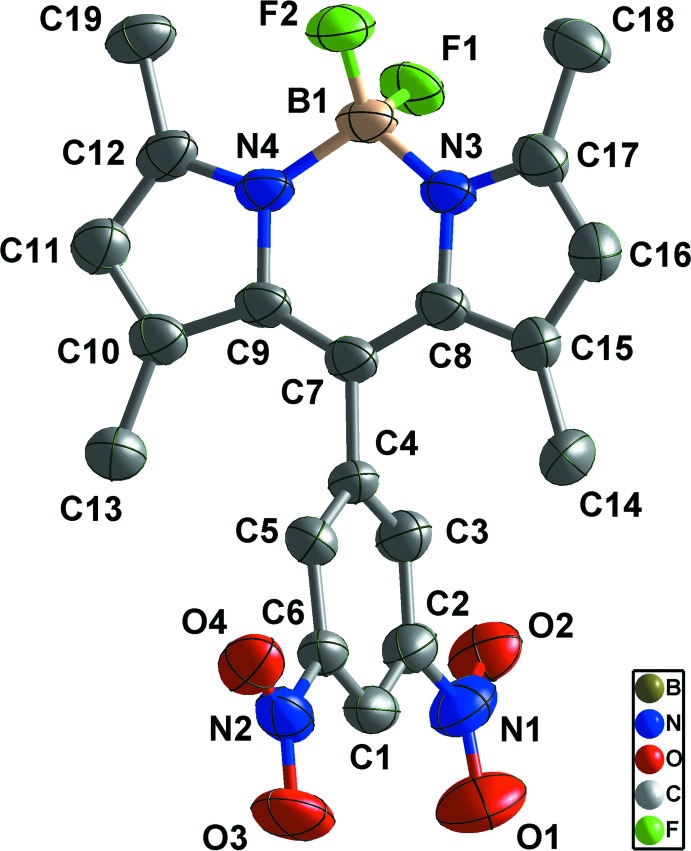
Molecular structure of the title compound with the atom-numbering scheme. Displacement ellipsoids are drawn at the 30% probability level. Hydrogen atoms are omitted for clarity.

**Fig. 2.**
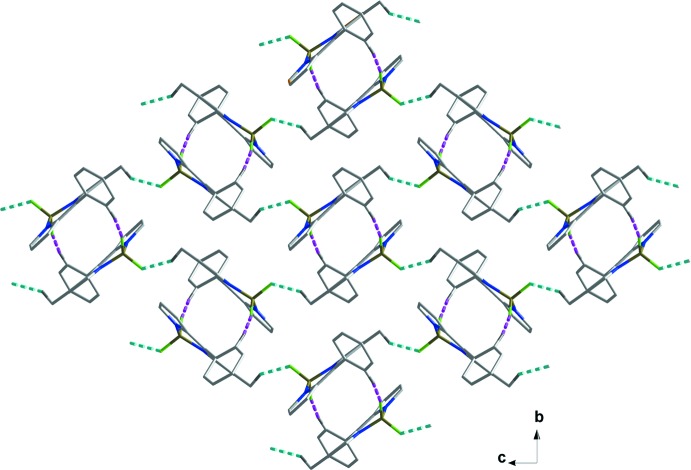
View of the two-dimensional supramolecular layered structure constructed via intermolecular C—H···F hydrogen bonding interactions. Nitro groups and irrelevant hydrogen atoms are omitted for clarity.

## supplementary crystallographic information

### Comment

In the past decades, many novel boron-dipyrromethene (BODIPY) dyes were developed for fluorescence analysis (Weiner *et al.*, 2001; Gabe *et al.*, 2004) and their crystal structures were investigated at the same time (Euler *et al.*, 2002*a*,*b*). As part of our ongoing studies of the substituent effect on the solid-state structures of BODIPY derivatives (Cui *et al.*, 2006), we report herein the crystal strcuture of the title compound, 4,4-difluoro-1,3,5,7-tetramethyl-8-(3',5'-dinitro-phenyl)-4-bora-3a,4a-diaza-*s*-indacene, (I).

A perspective view of (I), including the atomic numbering scheme, is shown in Fig. 1. The bond lengths and angles are within normal ranges. In the asymmetric unit, the BODIPY fragment is nearly planar, with a maximum deviation from the least-squares plane of 0.251 (2) Å. Owing to the steric hindrance, the benzene ring is almost perpendicular to the boron-dipyrromethene mean plane with a dihedral angle of 86.8 (6)° between the mean planes of the BODIPY fragment and the benzene ring. Intermolecular C5—H5···F2 (Table 1) hydrogen bonding connects two neighbouring molecules into a centrosymmetric dimer, which is further linked by another strong C—H···F interaction (C13—H13A···F1, Table 1) to form a supramolecular layered array, as depicted in Fig. 2.

### Experimental

Compound (I) was synthesized by the reaction of 2,4-dimethylpyrrole with 3,5-dinitro-benzaldehyde and boron trifluoride in a one-pot reaction (Kollmannsberger *et al.*, 1998). General procedure: 4.2 mmol of 2,4-dimethylpyrrole and 2 mmol of the aldehyde were dissolved in 200 ml of absolute methylene chloride under nitrogen atmosphere. One drop of trifluoroacetic acid was added and the solution was stirred at room temperature until TLC-control showed complete consumption of the aldehyde. At this point, 2 mmol dichlorodicyanobenzoquinone (DDQ) was added, and stirring was continued for 30 min followed by quick addition of 4 ml of triethylamine and 4 ml of boron trifluoride etherate. After stirring for another 3 h, the reaction mixture was washed with water and dried, and the solvent was evaporated. The residue was chromatographed twice on a silica column (a mixture of dichloromethane and hexane, *v*:*v*: 1:1, was used as the eluting solvent). Total yield: 42%. Purple crystals. ^1^H NMR (CDCl_3_): δ 1.46 (s, 6H, CH_3_), 1.96 (s, 6H, CH_3_), 5.62 (s, 2H, CH), 8.65 (s, 2H, CH), 8.90 (s, H, CH). MS (ESI), m/*z*: 414.1 [M—H]^-^. HRMS: [M—H]^-^ calculated: 414.176, measured: 414.143.

Purple single crystals suitable for X-ray analysis were obtained by dissolving (I) (0.15 g) in a hexane/dichloromethane (15 ml, *v*:*v*: 1:3) mixture and slowly evaporating the solvent at room temperature for a period of about one month.

### Refinement

All H atoms bound to C atoms were assigned to calculated positions, with C—H = 0.96 Å (methyl) and 0.93 Å (aromatic), and refined using a riding model, with *U*_iso_(H)=1.2U_eq_ (C).

### Crystal data

**Table:**

C_19_H_17_BF_2_N_4_O_4_	*F*(000) = 1712
*M**_r_* = 414.18	*D*_x_ = 1.434 Mg m^−^^3^
Monoclinic, *C*2/*c*	Mo *K*α radiation, λ = 0.71073 Å
Hall symbol: -C 2yc	Cell parameters from 2164 reflections
*a* = 29.016 (3) Å	θ = 2.4–24.3°
*b* = 9.1763 (9) Å	µ = 0.11 mm^−^^1^
*c* = 16.8294 (16) Å	*T* = 295 K
β = 121.086 (2)°	Block, purple
*V* = 3837.4 (7) Å^3^	0.21 × 0.21 × 0.16 mm
*Z* = 8	

### Data collection

**Table:**

Bruker APEXII CCD diffractometer	3767 independent reflections
Radiation source: fine-focus sealed tube	2276 reflections with *I* > 2σ(*I*)
Graphite monochromator	*R*_int_ = 0.035
φ and ω scans	θ_max_ = 26.0°, θ_min_ = 1.6°
Absorption correction: multi-scan (*SADABS*; Sheldrick, 2003)	*h* = −34→35
*T*_min_ = 0.973, *T*_max_ = 0.985	*k* = −9→11
11322 measured reflections	*l* = −20→18

### Refinement

**Table:**

Refinement on *F*^2^	Primary atom site location: structure-invariant direct methods
Least-squares matrix: full	Secondary atom site location: difference Fourier map
*R*[*F*^2^ > 2σ(*F*^2^)] = 0.045	Hydrogen site location: inferred from neighbouring sites
*wR*(*F*^2^) = 0.114	H-atom parameters constrained
*S* = 1.04	*w* = 1/[σ^2^(*F*_o_^2^) + (0.0507*P*)^2^] where *P* = (*F*_o_^2^ + 2*F*_c_^2^)/3
3767 reflections	(Δ/σ)_max_ < 0.001
275 parameters	Δρ_max_ = 0.19 e Å^−^^3^
0 restraints	Δρ_min_ = −0.19 e Å^−^^3^

### Special details

**Table:**

Geometry. All esds (except the esd in the dihedral angle between two l.s. planes) are estimated using the full covariance matrix. The cell esds are taken into account individually in the estimation of esds in distances, angles and torsion angles; correlations between esds in cell parameters are only used when they are defined by crystal symmetry. An approximate (isotropic) treatment of cell esds is used for estimating esds involving l.s. planes.
Refinement. Refinement of F^2^ against ALL reflections. The weighted R-factor wR and goodness of fit S are based on F^2^, conventional R-factors R are based on F, with F set to zero for negative F^2^. The threshold expression of F^2^ > 2sigma(F^2^) is used only for calculating R-factors(gt) etc. and is not relevant to the choice of reflections for refinement. R-factors based on F^2^ are statistically about twice as large as those based on F, and R- factors based on ALL data will be even larger.

### Fractional atomic coordinates and isotropic or equivalent isotropic displacement parameters (Å^2^)

**Table:**

	*x*	*y*	*z*	*U*_iso_*/*U*_eq_	
B1	0.21971 (10)	0.3620 (3)	0.35983 (14)	0.0507 (6)	
N1	0.02477 (8)	0.7770 (2)	0.44115 (15)	0.0721 (5)	
N2	0.10066 (8)	0.3996 (2)	0.68190 (12)	0.0618 (5)	
N3	0.16759 (6)	0.29249 (18)	0.34430 (10)	0.0501 (4)	
N4	0.24400 (6)	0.45079 (17)	0.45002 (10)	0.0481 (4)	
O1	−0.00805 (8)	0.8018 (2)	0.46364 (14)	0.1030 (6)	
O2	0.02809 (8)	0.8443 (2)	0.38261 (15)	0.1074 (7)	
O3	0.07160 (8)	0.44618 (19)	0.70880 (11)	0.0898 (6)	
O4	0.12896 (7)	0.2925 (2)	0.71305 (11)	0.0828 (5)	
C1	0.06408 (8)	0.5895 (2)	0.56281 (13)	0.0539 (5)	
H1	0.0419	0.6201	0.5843	0.065*	
C2	0.06252 (8)	0.6547 (2)	0.48802 (13)	0.0519 (5)	
C3	0.09496 (8)	0.6107 (2)	0.45427 (13)	0.0513 (5)	
H3A	0.0926	0.6562	0.4029	0.062*	
C4	0.13103 (7)	0.4974 (2)	0.49844 (11)	0.0445 (5)	
C5	0.13404 (8)	0.4307 (2)	0.57523 (12)	0.0468 (5)	
H5	0.1586	0.3562	0.6064	0.056*	
C6	0.09981 (8)	0.4773 (2)	0.60432 (12)	0.0474 (5)	
C7	0.16365 (7)	0.4434 (2)	0.45850 (11)	0.0451 (5)	
C8	0.13997 (8)	0.3386 (2)	0.38813 (12)	0.0493 (5)	
C9	0.21486 (8)	0.4977 (2)	0.49101 (12)	0.0450 (5)	
C10	0.24901 (8)	0.5908 (2)	0.56652 (12)	0.0478 (5)	
C11	0.29708 (8)	0.5971 (2)	0.56926 (13)	0.0544 (5)	
H11	0.3273	0.6492	0.6121	0.065*	
C12	0.29309 (8)	0.5123 (2)	0.49699 (13)	0.0524 (5)	
C13	0.23721 (9)	0.6657 (2)	0.63311 (13)	0.0596 (6)	
H13A	0.2356	0.5949	0.6735	0.089*	
H13B	0.2033	0.7155	0.5992	0.089*	
H13C	0.2652	0.7349	0.6693	0.089*	
C14	0.04881 (9)	0.2612 (3)	0.37558 (16)	0.0758 (7)	
H14A	0.0298	0.3520	0.3555	0.114*	
H14B	0.0651	0.2521	0.4416	0.114*	
H14C	0.0241	0.1821	0.3454	0.114*	
C15	0.09174 (8)	0.2570 (2)	0.35092 (13)	0.0563 (5)	
C16	0.09160 (9)	0.1637 (3)	0.28651 (13)	0.0636 (6)	
H16	0.0650	0.0956	0.2515	0.076*	
C17	0.13746 (9)	0.1876 (2)	0.28228 (13)	0.0572 (5)	
C18	0.15291 (10)	0.1167 (3)	0.21963 (14)	0.0707 (7)	
H18A	0.1544	0.1887	0.1797	0.106*	
H18B	0.1267	0.0440	0.1827	0.106*	
H18C	0.1876	0.0716	0.2561	0.106*	
C19	0.33455 (9)	0.4896 (3)	0.47106 (15)	0.0665 (6)	
H19A	0.3463	0.3900	0.4824	0.100*	
H19B	0.3647	0.5527	0.5076	0.100*	
H19C	0.3193	0.5116	0.4065	0.100*	
F1	0.20797 (5)	0.45458 (14)	0.28592 (7)	0.0708 (4)	
F2	0.25531 (5)	0.25610 (13)	0.36548 (8)	0.0695 (4)	

### Atomic displacement parameters (Å^2^)

**Table:**

	*U*^11^	*U*^22^	*U*^33^	*U*^12^	*U*^13^	*U*^23^
B1	0.0537 (15)	0.0672 (15)	0.0426 (12)	0.0197 (12)	0.0330 (11)	0.0136 (11)
N1	0.0556 (12)	0.0716 (13)	0.0916 (15)	0.0124 (10)	0.0396 (11)	0.0006 (11)
N2	0.0609 (13)	0.0867 (14)	0.0507 (10)	−0.0185 (11)	0.0379 (10)	−0.0127 (10)
N3	0.0512 (10)	0.0681 (11)	0.0365 (8)	0.0141 (9)	0.0266 (8)	0.0045 (8)
N4	0.0472 (10)	0.0679 (11)	0.0399 (8)	0.0095 (8)	0.0302 (8)	0.0095 (8)
O1	0.0840 (13)	0.1048 (15)	0.1403 (16)	0.0355 (11)	0.0721 (13)	0.0046 (12)
O2	0.0984 (15)	0.1041 (15)	0.1317 (16)	0.0395 (12)	0.0679 (13)	0.0494 (14)
O3	0.1098 (15)	0.1162 (14)	0.0872 (12)	−0.0138 (11)	0.0820 (11)	−0.0146 (10)
O4	0.0828 (12)	0.1060 (14)	0.0694 (10)	0.0068 (11)	0.0462 (9)	0.0238 (10)
C1	0.0446 (12)	0.0673 (14)	0.0607 (12)	−0.0095 (10)	0.0349 (10)	−0.0200 (11)
C2	0.0401 (11)	0.0577 (12)	0.0586 (12)	0.0029 (10)	0.0259 (10)	−0.0053 (10)
C3	0.0438 (12)	0.0643 (13)	0.0493 (11)	0.0043 (10)	0.0265 (10)	0.0031 (10)
C4	0.0372 (11)	0.0629 (12)	0.0381 (10)	0.0010 (9)	0.0227 (9)	−0.0028 (9)
C5	0.0422 (11)	0.0621 (12)	0.0385 (10)	0.0014 (9)	0.0226 (9)	−0.0045 (9)
C6	0.0443 (12)	0.0645 (13)	0.0398 (10)	−0.0092 (10)	0.0263 (9)	−0.0097 (10)
C7	0.0426 (11)	0.0635 (12)	0.0334 (10)	0.0109 (10)	0.0227 (9)	0.0072 (9)
C8	0.0438 (12)	0.0721 (13)	0.0368 (10)	0.0096 (10)	0.0242 (9)	0.0019 (10)
C9	0.0465 (12)	0.0617 (12)	0.0359 (9)	0.0090 (10)	0.0278 (9)	0.0091 (9)
C10	0.0517 (12)	0.0574 (12)	0.0416 (10)	0.0060 (10)	0.0293 (9)	0.0102 (9)
C11	0.0486 (13)	0.0685 (14)	0.0502 (11)	−0.0013 (10)	0.0285 (10)	0.0064 (10)
C12	0.0475 (13)	0.0694 (14)	0.0489 (11)	0.0071 (10)	0.0310 (10)	0.0171 (11)
C13	0.0604 (14)	0.0709 (14)	0.0540 (12)	−0.0030 (11)	0.0342 (11)	−0.0046 (11)
C14	0.0470 (13)	0.1140 (19)	0.0679 (14)	−0.0121 (13)	0.0308 (11)	−0.0276 (14)
C15	0.0429 (12)	0.0824 (15)	0.0398 (10)	0.0061 (11)	0.0186 (9)	−0.0057 (10)
C16	0.0509 (14)	0.0831 (16)	0.0476 (12)	0.0036 (12)	0.0189 (10)	−0.0102 (11)
C17	0.0602 (14)	0.0725 (14)	0.0369 (10)	0.0164 (12)	0.0235 (10)	0.0029 (11)
C18	0.0807 (17)	0.0867 (16)	0.0507 (12)	0.0180 (14)	0.0381 (12)	−0.0042 (12)
C19	0.0601 (14)	0.0842 (16)	0.0769 (14)	0.0077 (12)	0.0508 (12)	0.0157 (13)
F1	0.0903 (10)	0.0893 (9)	0.0471 (6)	0.0167 (7)	0.0457 (6)	0.0206 (6)
F2	0.0640 (8)	0.0823 (8)	0.0780 (8)	0.0215 (7)	0.0478 (7)	0.0026 (6)

### Geometric parameters (Å, º)

**Table:**

B1—F2	1.385 (2)	C7—C8	1.400 (3)
B1—F1	1.396 (2)	C8—C15	1.416 (3)
B1—N3	1.534 (3)	C9—C10	1.423 (3)
B1—N4	1.536 (3)	C10—C11	1.373 (3)
N1—O2	1.208 (2)	C10—C13	1.497 (2)
N1—O1	1.214 (2)	C11—C12	1.397 (3)
N1—C2	1.480 (3)	C11—H11	0.9300
N2—O4	1.213 (2)	C12—C19	1.491 (3)
N2—O3	1.222 (2)	C13—H13A	0.9600
N2—C6	1.477 (2)	C13—H13B	0.9600
N3—C17	1.356 (3)	C13—H13C	0.9600
N3—C8	1.405 (2)	C14—C15	1.503 (3)
N4—C12	1.345 (2)	C14—H14A	0.9600
N4—C9	1.407 (2)	C14—H14B	0.9600
C1—C6	1.369 (3)	C14—H14C	0.9600
C1—C2	1.373 (3)	C15—C16	1.380 (3)
C1—H1	0.9300	C16—C17	1.386 (3)
C2—C3	1.387 (2)	C16—H16	0.9300
C3—C4	1.387 (3)	C17—C18	1.492 (3)
C3—H3A	0.9300	C18—H18A	0.9600
C4—C5	1.392 (2)	C18—H18B	0.9600
C4—C7	1.500 (2)	C18—H18C	0.9600
C5—C6	1.383 (2)	C19—H19A	0.9600
C5—H5	0.9300	C19—H19B	0.9600
C7—C9	1.385 (3)	C19—H19C	0.9600
			
F2—B1—F1	108.56 (15)	N4—C9—C10	107.70 (17)
F2—B1—N3	110.77 (18)	C11—C10—C9	106.38 (16)
F1—B1—N3	109.62 (16)	C11—C10—C13	125.20 (19)
F2—B1—N4	111.02 (16)	C9—C10—C13	128.40 (18)
F1—B1—N4	109.52 (18)	C10—C11—C12	108.75 (18)
N3—B1—N4	107.34 (14)	C10—C11—H11	125.6
O2—N1—O1	124.3 (2)	C12—C11—H11	125.6
O2—N1—C2	118.27 (19)	N4—C12—C11	109.37 (17)
O1—N1—C2	117.4 (2)	N4—C12—C19	122.61 (19)
O4—N2—O3	124.47 (19)	C11—C12—C19	128.0 (2)
O4—N2—C6	118.18 (17)	C10—C13—H13A	109.5
O3—N2—C6	117.3 (2)	C10—C13—H13B	109.5
C17—N3—C8	107.57 (17)	H13A—C13—H13B	109.5
C17—N3—B1	127.84 (16)	C10—C13—H13C	109.5
C8—N3—B1	124.36 (17)	H13A—C13—H13C	109.5
C12—N4—C9	107.79 (16)	H13B—C13—H13C	109.5
C12—N4—B1	127.57 (16)	C15—C14—H14A	109.5
C9—N4—B1	124.17 (16)	C15—C14—H14B	109.5
C6—C1—C2	116.96 (17)	H14A—C14—H14B	109.5
C6—C1—H1	121.5	C15—C14—H14C	109.5
C2—C1—H1	121.5	H14A—C14—H14C	109.5
C1—C2—C3	122.70 (19)	H14B—C14—H14C	109.5
C1—C2—N1	118.96 (18)	C16—C15—C8	106.07 (18)
C3—C2—N1	118.34 (18)	C16—C15—C14	124.5 (2)
C2—C3—C4	118.85 (18)	C8—C15—C14	129.41 (18)
C2—C3—H3A	120.6	C15—C16—C17	109.2 (2)
C4—C3—H3A	120.6	C15—C16—H16	125.4
C3—C4—C5	119.71 (16)	C17—C16—H16	125.4
C3—C4—C7	118.93 (15)	N3—C17—C16	109.11 (17)
C5—C4—C7	121.26 (17)	N3—C17—C18	123.0 (2)
C6—C5—C4	118.72 (18)	C16—C17—C18	127.9 (2)
C6—C5—H5	120.6	C17—C18—H18A	109.5
C4—C5—H5	120.6	C17—C18—H18B	109.5
C1—C6—C5	123.04 (18)	H18A—C18—H18B	109.5
C1—C6—N2	118.49 (17)	C17—C18—H18C	109.5
C5—C6—N2	118.45 (19)	H18A—C18—H18C	109.5
C9—C7—C8	122.66 (16)	H18B—C18—H18C	109.5
C9—C7—C4	120.02 (17)	C12—C19—H19A	109.5
C8—C7—C4	117.32 (16)	C12—C19—H19B	109.5
C7—C8—N3	119.15 (17)	H19A—C19—H19B	109.5
C7—C8—C15	132.72 (17)	C12—C19—H19C	109.5
N3—C8—C15	108.06 (17)	H19A—C19—H19C	109.5
C7—C9—N4	119.35 (17)	H19B—C19—H19C	109.5
C7—C9—C10	132.80 (16)		
			
F2—B1—N3—C17	−47.3 (2)	C9—C7—C8—C15	172.3 (2)
F1—B1—N3—C17	72.5 (2)	C4—C7—C8—C15	−8.1 (3)
N4—B1—N3—C17	−168.62 (16)	C17—N3—C8—C7	177.55 (16)
F2—B1—N3—C8	138.92 (17)	B1—N3—C8—C7	−7.6 (3)
F1—B1—N3—C8	−101.31 (19)	C17—N3—C8—C15	0.4 (2)
N4—B1—N3—C8	17.6 (2)	B1—N3—C8—C15	175.24 (16)
F2—B1—N4—C12	48.5 (3)	C8—C7—C9—N4	2.4 (3)
F1—B1—N4—C12	−71.4 (2)	C4—C7—C9—N4	−177.16 (15)
N3—B1—N4—C12	169.67 (17)	C8—C7—C9—C10	−172.43 (19)
F2—B1—N4—C9	−140.44 (17)	C4—C7—C9—C10	8.0 (3)
F1—B1—N4—C9	99.7 (2)	C12—N4—C9—C7	−176.52 (16)
N3—B1—N4—C9	−19.2 (2)	B1—N4—C9—C7	10.9 (3)
C6—C1—C2—C3	0.4 (3)	C12—N4—C9—C10	−0.5 (2)
C6—C1—C2—N1	−179.64 (17)	B1—N4—C9—C10	−173.08 (16)
O2—N1—C2—C1	171.6 (2)	C7—C9—C10—C11	175.1 (2)
O1—N1—C2—C1	−9.3 (3)	N4—C9—C10—C11	−0.2 (2)
O2—N1—C2—C3	−8.4 (3)	C7—C9—C10—C13	−3.3 (3)
O1—N1—C2—C3	170.7 (2)	N4—C9—C10—C13	−178.58 (18)
C1—C2—C3—C4	−0.9 (3)	C9—C10—C11—C12	0.8 (2)
N1—C2—C3—C4	179.11 (17)	C13—C10—C11—C12	179.24 (18)
C2—C3—C4—C5	0.0 (3)	C9—N4—C12—C11	1.0 (2)
C2—C3—C4—C7	176.17 (17)	B1—N4—C12—C11	173.25 (17)
C3—C4—C5—C6	1.5 (3)	C9—N4—C12—C19	−178.41 (17)
C7—C4—C5—C6	−174.66 (17)	B1—N4—C12—C19	−6.1 (3)
C2—C1—C6—C5	1.1 (3)	C10—C11—C12—N4	−1.1 (2)
C2—C1—C6—N2	−176.91 (16)	C10—C11—C12—C19	178.22 (19)
C4—C5—C6—C1	−2.1 (3)	C7—C8—C15—C16	−176.2 (2)
C4—C5—C6—N2	175.98 (16)	N3—C8—C15—C16	0.4 (2)
O4—N2—C6—C1	173.66 (18)	C7—C8—C15—C14	1.6 (4)
O3—N2—C6—C1	−4.6 (3)	N3—C8—C15—C14	178.3 (2)
O4—N2—C6—C5	−4.5 (3)	C8—C15—C16—C17	−1.0 (2)
O3—N2—C6—C5	177.23 (17)	C14—C15—C16—C17	−179.0 (2)
C3—C4—C7—C9	94.5 (2)	C8—N3—C17—C16	−1.0 (2)
C5—C4—C7—C9	−89.4 (2)	B1—N3—C17—C16	−175.65 (17)
C3—C4—C7—C8	−85.2 (2)	C8—N3—C17—C18	177.46 (18)
C5—C4—C7—C8	91.0 (2)	B1—N3—C17—C18	2.8 (3)
C9—C7—C8—N3	−4.0 (3)	C15—C16—C17—N3	1.3 (2)
C4—C7—C8—N3	175.56 (16)	C15—C16—C17—C18	−177.1 (2)

### Hydrogen-bond geometry (Å, º)

**Table:**

*D*—H···*A*	*D*—H	H···*A*	*D*···*A*	*D*—H···*A*
C5—H5···F2^i^	0.93	2.51	3.307 (2)	144
C13—H13*A*···F1^ii^	0.96	2.45	3.287 (2)	146

Symmetry codes: (i) −*x*+1/2, −*y*+1/2, −*z*+1; (ii) *x*, −*y*+1, *z*+1/2.
